# Arsenic Inhibits Proliferation and Induces Autophagy of Tumor Cells in Pleural Effusion of Patients with Non-Small Cell Lung Cancer Expressing EGFR with or without Mutations via PI3K/AKT/mTOR Pathway

**DOI:** 10.3390/biomedicines11061721

**Published:** 2023-06-15

**Authors:** Jianhua Mao, Xiaoqian Shi, Li Hua, Menghang Yang, Yan Shen, Zheng Ruan, Bing Li, Xiaodong Xi

**Affiliations:** 1 Shanghai Institute of Hematology, State Key Laboratory of Medical Genomics, Collaborative Innovation Center of Hematology, Ruijin Hospital, Shanghai Jiao Tong University School of Medicine, Shanghai 200025, China; maojianhua110@163.com (J.M.); ruan609@126.com (Z.R.); 2 Department of Respiratory and Critical Care Medicine, Shanghai Fourth People’s Hospital, School of Medicine, Tongji University, 1279 Sanmen Road, Shanghai 200434, China; shixiaoqian0210@163.com; 3 School of Public Health, Shanghai Jiao Tong University School of Medicine, Shanghai 200025, China; seyhuali@163.com; 4 Department of Oncology, Shanghai Pulmonary Hospital, School of Medicine, Tongji University, 507 Zhengmin Road, Shanghai 200433, China; yangmenghang@tongji.edu.cn; 5 Research Center for Experimental Medicine, Ruijin Hospital, Shanghai Jiao Tong University School of Medicine, Shanghai 200025, China; sy40685@rjh.com.cn; 6 Department of Respiratory and Critical Care Medicine, Changzheng Hospital, Naval Medical University, 415 Fengyang Road, Shanghai 200003, China

**Keywords:** arsenic, gefitinib, non-small cell lung cancer, pleural effusion, autophagy

## Abstract

To clarify whether arsenic could exert inhibitory effects on tumor cells in pleural effusions of patients with non-small cell lung cancer (NSCLC), 36 NSCLC pleural effusion samples were collected from Changzheng Hospital and Ruijin Hospital, from 2019 to 2022. The genotype of epidermal growth factor receptor (*EGFR*) was identified. Tumor cells were isolated and treated with arsenic trioxide (ATO) or/and gefitinib. Additionally, six patients were intrapleurally administrated with ATO. Results showed that 25 samples bore *EGFR* wild type (WT) and 11 harbored *EGFR* mutations, including 6 with L858R, 3 with ΔE746-A750, and 2 with T790M. ATO diminished the number of tumor cells from patients with WT and mutant *EGFR*, down-regulated the expression or phosphorylation of EGFR, pmTOR, PI3K, PTEN, and p4E-BP1, and up-regulated the expression of LC3. Immunofluorescent experiments showed that ATO enhanced LC3 and P62. By contrast, gefitinib was only effective in those harboring *EGFR* sensitizing mutations. Notably, in patients with intrapleural ATO injection, the pleural effusion underwent a bloody to pale yellow color change, the volume of the pleural effusion was reduced, and the number of the tumor cells was significantly reduced. In conclusion, arsenic is effective against NSCLC with various *EGFR* genotypes in vitro and in vivo, and potentially circumvents gefitinib resistance.

## 1. Introduction

Lung cancer is one of the most common malignant tumors and remains the leading cause of cancer-related mortality, accounting for about 2.2 million new cases and 1.8 million deaths worldwide every year [1,2]. About 57% of lung cancer cases have metastasis at the diagnostic stage, and the overall 5-year survival rate is merely 5% [3]. Histologically, lung cancer is divided into small cell lung cancer (SCLC) and non-small cell lung cancer (NSCLC), of which NSCLC accounts for approximately 85% [4]. Recently, NSCLC was recognized as a heterogeneous disease at the molecular level, so the newly diagnosed advanced stage NSCLC specimen is recommended to be tested for predictive biomarkers [5]. Testing is recommended for sensitizing epidermal growth factor receptor (*EGFR*) mutation, anaplastic lymphoma kinase (*ALK*) fusions, ROS proto-oncogene 1 (*ROS1*) fusions, B-Raf proto-oncogene (*BRAF*) V600E, neurotrophic tropomyosin-receptor kinase (*NTRK*) fusions, rearranged during transfection (*RET*) fusions, and mesenchymal epithelial transition factor (*MET*) exon 14 skipping alterations, as well as programmed cell death protein-ligand 1 (PD-L1) immunohistochemistry (IHC), given that there are highly effective targeted therapies that have been approved for these targets [5,6,7]. In addition, *EGFR* exon 20 insertions, Erb-B2 receptor tyrosine kinase 2 (*ERBB2*) mutations, neuregulin 1 (*NRG1*) fusions, *KRAS* proto-oncogene (*KRAS*) G12C, and tumor mutational burden (TMB) are evolving targeting/biomarkers and may eventually be included in the test panels, if possible [6,7]. The treatment of lung cancer has also evolved with the introduction of several lines of tyrosine kinase inhibitors (TKIs) in patients with *EGFR*, *ALK*, *ROS1*, and *NTRK* mutations [8]. Similarly, immune checkpoint inhibitors (ICIs) have dramatically changed the landscape of NSCLC treatment. ICIs are now part of the first-line NSCLC treatment strategy as monotherapy, combined with chemotherapy, or after definite chemotherapy in patients with stage III unresectable NSCLC accompanied by PD-L1 expression [8,9]. Further clinical trials would be helpful to understand the role of these novel agents and the extent to which the patients will benefit from them [8].

Among the biomarkers, abnormal activation caused by high expression or mutation of *EGFR* plays a driving role in the pathogenesis of NSCLC [10]. About 50% of Asian NSCLC patients harbor *EGFR* sensitizing mutations, including L858R in exon 21 or E746-A750 deletion mutations (ΔE746-A750) in exon 19, which are sensitive to EGFR TKIs, such as gefitinib and erlotinib [11]. Although gefitinib has been approved by the Food and Drug Administration (FDA) of USA as a first-line drug for the treatment of EGFR-activated mutants NSCLC [12], acquired resistance and recurrence are inevitable in most patients 9–13 months after treatment, and genetic testing indicated that the T790M mutation in exon 20 is the main cause of drug resistance [13]. Researchers designed a third-generation TKI (osimertinib) to overcome the T790M mutation and achieved good efficacy [14], but after a period of treatment, the third mutation C797S appeared, resulting in acquired resistance to osimertinib [15]. Although new TKIs (such as EAI045 [16] and JBJ-04-125-02 [17]) have been developed, new mutations may still be screened and they were selected under the action of drug treatment, leading to a process of tumor evolution caused by acquired drug resistance [18]. Therefore, a novel strategy to overcome TKI resistance and improve their therapeutic efficacy for NSCLC is urgently required.

As an ancient Chinese medicine, arsenic enabled the 7-year disease-free survival (DFS) rate of acute promyelocytic leukemia (APL) to reach 95.7% when combined with all-trans retinoic acid [19]. The long-term efficacy and safety of arsenic trioxide (ATO) in the treatment of APL at an intravenous dose of 0.16 mg/kg (10 mg maximum) has been demonstrated in previous studies [19,20]. In the treatment of solid tumors, including NSCLC, our previous published study revealed that arsenic trioxide (ATO) at 2 μmol/L can suppress the proliferation of NSCLC cell lines, especially the gefitinib-resistant NCI-H1975 cells, and ATO at the dose of 5 mg/kg/d can inhibit the tumor growth of subcutaneous tumors and carcinoma in situ in mice [21]. The molecular mechanisms of arsenic to treat NSCLC involve the processes where arsenic directly binds to P62, confirmed by the interaction of biotinylated arsenic with P62, by partial colocalization of ReAsH (a fluorescent, membrane-permeable biarsenical compound) with P62 in immunofluorescent (IF) assays, and by binding of ATO to His-P62 in surface plasmon resonance (SPR) assays at a KD value of 8.76 nmol/L; mediates the conformational changes of P62; and enhances the interaction of P62 and EGFR, preferentially the L858R/T790M-mutated EGFR, leading to an autophagy-based EGFR degradation [21]. Recent research has demonstrated that arsenic sulfide could reverse cisplatin resistance in NSCLC in vitro and in vivo through targeting PD-L1 [22]. Pleural effusion often appears in advanced NSCLC patients with pleural metastasis [23]. Previous studies demonstrated that the intrapleural administration of arsenic reduced the amount of pleural effusion and lightened the color of the pleural effusion from black-red to pale yellow [24,25], suggesting a reduction in the permeability of blood vessels and the leakage of red blood cells. The overall remission (OR) is more than 70% [24,25]. However, the effects and the underlying mechanisms of arsenic alone or in combination with gefitinib on tumor cells in pleural effusion still need to be elucidated.

In this study, we focused on the inhibitory effects of arsenic trioxide (ATO) and gefitinib alone or in combination on tumor cells in pleural effusion, the involved pathways, and the underlying mechanisms, which can provide important insights into novel therapeutic strategies for NSCLC.

## 2. Materials and Methods

### 2.1. Subjects, Reagents, and Instruments

#### 2.1.1. Subjects

Thirty-six pleural effusion specimens from NSCLC patients were collected from the Department of Respiratory and Critical Care Medicine of the Changzheng Hospital affiliated to Naval Medical University, and the Department of Laboratory and Pathology of the Ruijin Hospital affiliated to Shanghai Jiao Tong University School of Medicine, from January 2019 to January 2022. Twenty-three males and thirteen females were enrolled. The median age of overall patients was 63.5 [54.0, 73.0] years old. All cases were confirmed by cytology and/or pathology, and the basic information of the gender, age, smoking status, type of pathology, tumor TNM stage, appearance of pleural effusions, and EGFR genotype was collected and is shown in Table 1. The experiment was approved by the Ethics Committee of Changzheng Hospital affiliated to the Naval Medical University (2015-017), and by the Ethics Committee of Ruijin Hospital affiliated to Shanghai Jiao Tong University School of Medicine (2022-228). This work complied with the principles laid down in the Declaration of Helsinki. The informed consent was obtained from all subjects involved in the study.

#### 2.1.2. Reagents and Instruments

Fetal bovine serum (FBS), antibiotic (antimycolic 100×), and 0.25% trypsin-EDTA were purchased from GIBCO (Thermo Fisher Scientific, Waltham, MA, USA). DMEM medium and phosphate buffer (PBS) were purchased from Basalmedia Technologies Co., Ltd. (Shanghai, China). Wright’s staining solution was purchased from Shanghai Beyotime Biotechnology Co., Ltd. (Shanghai, China), while the blood/body fluid DNA extraction kit was purchased from TIANGEN (Shanghai, China). Polymerase chain reaction (PCR) amplification primers and sequencing primers were synthesized by BioSune Biotechnology Co., Ltd. (Shanghai, China). The immunological reagents included the antibodies (Abs) against phosphorylated mammalian target of rapamycin (pmTOR), phosphatidylinositol 3 kinase (PI3K), gene of phosphate and tension homology deleted on chromosome ten (PTEN), and phosphorylated eukaryotic initiation factor 4E binding protein 1 (p4E-BP1) which were purchased from Cell signaling technology (Danvers, MA, USA), and the Abs against EGFR, LC3, and P62 which were purchased from Abcam (Cambridge, UK), Santa Cruz Biotechnology (Dallas, TX, USA), and Medical and Biological Laboratories (Nagoya, Japan), respectively. Additionally, β-actin Ab was purchased from Sigma-Aldrich (Saint Louis, MO, USA), while horseradish peroxidase (HRP)-labeled sheep anti-rabbit Ab and HRP-labeled art anti-mouse Ab were purchased from Abcam (Cambridge, United Kingdom). Lymphocyte isolation solution was purchased from Shanghai Sangon Biotech (Shanghai, China), the protein quantification kit (Bicinchoninic acid (BCA) Assay) from Pierce (Appleton, WI, USA), Polyvinylidene Fluoride (PVDF) membrane from GE-Healthcare (Chicago, IL, USA), and HRP-labeled chromogenic substrate from Millipore (Burlington, MA, USA).

ATO was purchased from Beijing Shuang Lu (SL) Pharmaceutical Co., Ltd. (Beijing, China) and was prepared as a 2 mmol/L stock solution in PBS at −20 °C for in vitro experiments. Gefitinib was purchased from Sigma-Aldrich (St. Louis, MO, USA) and was dissolved in dimethylsulfoxide (DMSO) to produce a stock solution of 10 mmol/L at −20 °C.

The instruments used in this study included an inverted fluorescence microscope (Nikon Corporation, Tokyo, Japan), a laser confocal microscope SP8 MP (Leica Corporation, Hillsboro, OR, USA), 5% CO_2_ mixture cell incubator (Thermo Fisher Scientific Corporation, Waltham, MA, USA), cell smear centrifuge (CYTOPRO) (WESCOR Corporation, Stoneham, MA, USA), and Western blot (WB) developer LAS-4000 (FujiFilm, Tokyo, Japan).

### 2.2. Methods

#### 2.2.1. Collection of Pleural Effusion and Intrapleural Administration

After B-ultrasound examination and localization, the patient was placed under local anesthesia with 8–14 F (French gauge system) deep venous catheter drainage, and the external end was connected with a closed drainage bag. The initial drainage volume was less than 800 mL, followed by slow drainage of 1000–1500 mL. The pleural effusion was drained as much as possible before intrapleural administration at 200 mL/d (no negative pressure suction). To treat NSCLC patients, 40 mL of saline with 10 mg of ATO was injected into the pleural cavity, and the tube was clamped after injection. The patients were asked to rotate the body position so that the drug could widely and evenly distribute within the pleura. After 24 h, the pleural fluid was released and then drained again. When the pleural effusion was less than 200 mL/d, the drainage tube was removed. The procedure was based on what previously performed [24,25].

#### 2.2.2. Tumor Cell Isolation from Pleural Effusion and Wright’s Staining

The pleural effusion was collected and stored in a 500 mL glass bottle. The pleural effusion in the glass bottle was photographed to facilitate the monitoring of the volume and color. Following standing for 2 h, the pleural effusion was centrifuged at 800× *g* for 10 min, and the cells in the pleural effusion were collected. Then, the cells were re-suspended in PBS, the mononuclear cells (including tumor cells) were separated with lymphocyte separation solution, and the red blood cells and other cells were removed. The isolated primary tumor cells were counted using a cell counting plate, and the cell density was adjusted to 2 × 10^5^/mL. A quantity of 200 μL of cells was centrifuged by a cell smear centrifuge at 100× *g* for 5 min. The cells were evenly coated on the slide, and Wright’s staining solution was evenly dropped onto the cells for about 5 min. The cell staining was observed under a microscope. The staining was terminated when the results were satisfactory, and the cells were photographed.

#### 2.2.3. PCR and Sequencing

The DNA of the tumor cells (5 × 10^6^–1 × 10^7^) derived from pleural effusion was extracted according to the protocol provided by the blood/body fluid DNA extraction kit. Specific primers for EGFR exon 18, 19, 20, and 21 were used for PCR reactions. Primer information is shown as follows: (i) exon 18F: 5′-TCCAGCATGGTGAGGGCTGAG-3′ and 18R: 5′-GGCTCCCCACCAGACCATG-3′; (ii) exon 19F: 5′-TGGGCAGCATGTGGCACCATC-3′ and 19R: 5′-AGGTGGGCCTGAGGTTCAG-3′; (iii) exon 20F: 5′-CCTCCTT-CTGGCCACCATGCG-3′ and 20R: 5′-CATGTGAGGATCCTGGCTCC-3′; (iv) exon 21F: 5′-CGGATGCAGAGCTTCTTCCC-3′ and 21R: 5′-AGGCAGCCTGGTCCCTGGTG-3′. PCR products were detected by agarose electrophoresis before sequencing by BioSune. Sequencing primers were the same as PCR amplification primers. Forward and reverse sequencing were performed. Sequencing results were compared with wild type (WT) EGFR sequences to determine the EGFR genotype for each sample. The following PCR reaction conditions were adopted: 94 °C for 3 min, followed by 41 cycles of amplification of 94 °C for 40 s, 60 °C for 40 s, and 72 °C for 35 s, with a final extension at 72 °C for 5 min. The amplified fragment sizes were 242, 217, 296, and 275 bp for exon 18, 19, 20, and 21, respectively.

#### 2.2.4. Cell Culture and Drug Treatment

The tumor cells (5 × 10^6^–1 × 10^7^) isolated from pleural effusion were resuspended in DMEM medium containing 10% of FBS with 1% antibiotics (antibacterial/antifungal) together. According to the counts of tumor cells, the cells were seeded in 6-well plates or 60 mm cell culture dishes. The tumor cells isolated from each patient were seeded into 4 wells or 4 dishes, and then treated with ATO (2 μmol/L) alone, gefitinib (5 μmol/L) alone, or ATO combined with gefitinib. Cells were harvested at the 4th day after drug treatment. Wright’s staining, WB, and IF were performed.

#### 2.2.5. WB Assay

The total cells (5 × 10^6^) were lysed using RIPA lysate (50 mmol/L Tris, pH 8.0, 150 mmol/L NaCl, 0.1% SDS, 0.5% sodium deoxycholate, 1% NP-40) on ice for 30 min. After lysis, the cells were centrifuged at 13,400× *g* for 10 min, and the cell lysis supernatant was collected. The concentration of protein was quantified using a BCA quantification kit. After SDS-PAGE electrophoresis, the proteins were transferred to PVDF membrane and incubated with the indicated antibodies, and the corresponding secondary antibodies labeled with HRP. After incubation, HRP substrate was used for the development and LAS-4000 was used for photo capture.

#### 2.2.6. IF Assay

The autoclaved cover plates were placed into the wells of a 6-well plate, and then the cells were seeded into the wells. The same drug treatment scheme described above was followed. After four days of post-treatment, the cell medium was removed, and the cells were washed with PBS and then fixed with 4% paraformaldehyde. After that, 0.2% Triton X-100 was added to punch holes in the cell membrane to allow the antibodies to enter the cells. After blocking with 1% fetal bovine serum albumin (BSA), anti-LC3 and anti-P62 antibodies were added for incubation overnight. After washing with PBS 3 times, the Alexa Fluor 488-labeled anti-mouse antibody (the LC3 antibody was mouse monoclonal antibody) and the Alexa Fluor 647-labeled anti-rabbit antibody (the P62 antibody was rabbit polyclonal antibody) were added for incubation, and then the cells were washed with PBS 3 times. The cells were sealed by adding a DAPI-containing sealing solution. A laser confocal microscope, SP8 MP, was used for observation and photo capture.

#### 2.2.7. Statistical Analysis

Data are presented as mean ± standard deviation (SD). Sample size (*n*) is shown in each statistical result. Pairwise comparisons were performed using the student’s *t* test and two-tailed analysis (GraphPad Prism software, Ver 8.0.1, La Jolla, CA, USA), and one-way analysis of variance (ANOVA) was adopted with normally distributed data assuming equal variances (SPSS software, Ver 18, SPSS Inc., Chicago, IL, USA). * *p* < 0.05, ** *p* < 0.01, *** *p* < 0.001.

## 3. Results

### 3.1. Identification of the EGFR Genotype of Tumor Cells Derived from NSCLC Patients’ Pleural Effusion

**Figure 1:**
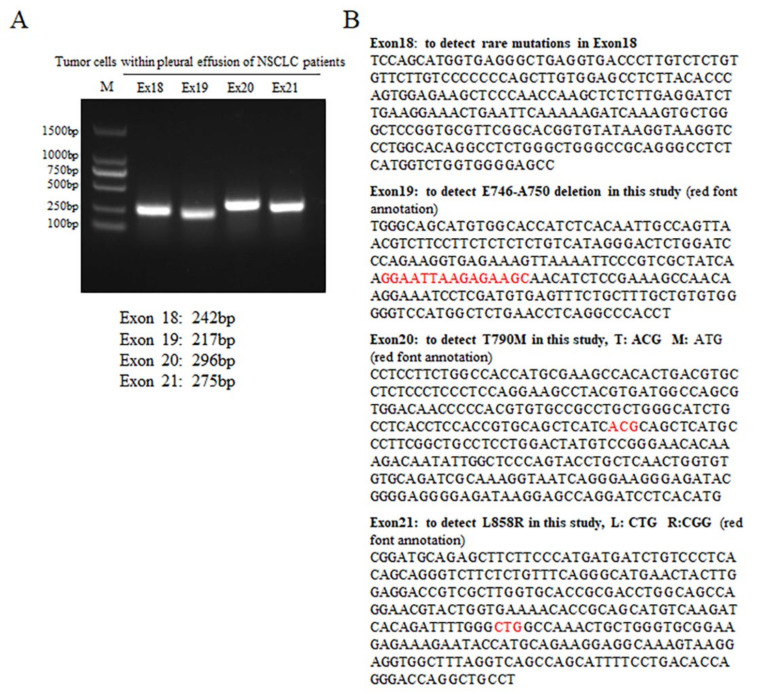
Identification of EGFR genotype in tumor cells derived from pleural effusions of NSCLC patients. (**A**). Agarose electrophoresis results of the PCR products of tumor cells using specific primers for EGFR Ex18, Ex19, Ex20, and Ex21. (**B**). Nucleotide sequences of the Ex18, Ex19, Ex20, and Ex21.

The PCR and agarose electrophoresis results for *EGFR* exon 18, 19, 20, and 21 are shown in Figure 1A. The PCR products had clear bands, and the fragment length was as expected. The PCR products were sent for sequencing. The sequencing results were compared with the gene sequences of exon 18, 19, 20 and 21, as shown in Figure 1B, to identify the *EGFR* genotype of each specimen. The basic information of the 36 NSCLC patients and the *EGFR* genotype results are exhibited in Table 1. There were 25 patients harboring *EGFR* WT, 6 patients carrying *EGFR* L858R mutation, and 3 patients harboring *EGFR* ΔE746-A750 mutation. The *EGFR* T790M mutation was present in two patients. *EGFR* WT, gefitinib-sensitive mutants, and gefitinib-resistant mutants genotypes were found in the overall collected samples.

### 3.2. In Vitro Effects of Arsenic and Gefitinib on Primary Tumor Cells Derived from NSCLC Patients’ Pleural Effusion

**Figure 2:**
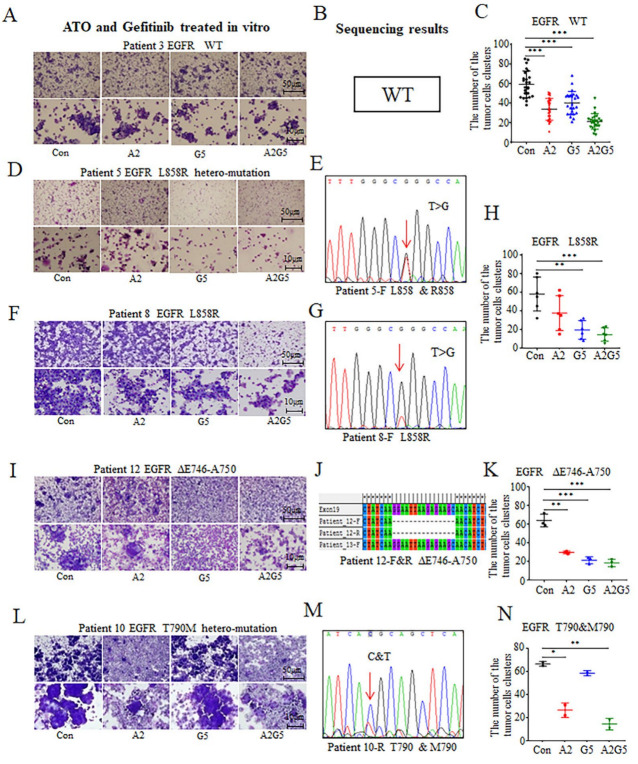
Inhibitory effects of arsenic and gefitinib on tumor cells derived from NSCLC patients with different EGFR genotypes. (**A**) Representative images of the effects of arsenic and gefitinib on tumor cells with EGFR WT. (**B**) Sequencing results. (**C**) Statistical graphs of the number of tumor cell clusters with or without drug treatment. Mean and standard deviation are shown. (**D**) Representative images of the effects of arsenic and gefitinib on tumor cells with EGFR L858R hetero-mutation. (**E**) Sequencing results. (**F**) Representative images of the effects of arsenic and gefitinib on tumor cells with EGFR L858R homo-mutation. (**G**) Sequencing results. (**H**) Statistical graph related to the effects of the drugs on EGFR L858R mutation. (**I**) Representative images of the effects of arsenic and gefitinib on tumor cells with EGFR ΔE746-A750. (**J**) Sequencing results. (**K**) Statistical graph. (**L**) Representative images of the effects of arsenic and gefitinib on tumor cells with EGFR T790M. (**M**) Sequencing results. (**N**) Statistical graph. Images were captured by an optical microscope. (Scale bars shown on the images). * *p* < 0.05, ** *p* < 0.01, *** *p* < 0.001. “Con” stands for “Control”. The tumor cells in the Con group were not treated with any drug. “A2” represents “ATO (2 μmol/L) alone”. The tumor cells in the A2 group were treated with 2 μmol/L arsenic trioxide alone. “G5” stands for “gefitinib (5 μmol/L) alone”. The tumor cells in the G5 group were treated with 5 μmol/L gefitinib. “A2G5” represents “ATO (2 μmol/L) combined with gefitinib (5 μmol/L)”. The tumor cells in the A2G5 group were treated with 2 μmol/L arsenic trioxide and 5 μmol/L gefitinib together.

Results showed that arsenic exerted an inhibitory effect on the primary tumor cells harboring EGFR WT (Figure 2A–C), L858R (Figure 2D–H), ΔE746-A750 (Figure 2I–K), and T790M genotypes (Figure 2L–N). ATO significantly diminished the number of clusters of tumor cells derived from the pleural effusions of NSCLC patients who harbored all kinds of EGFR genotypes (*p* < 0.001, *p* < 0.01, *p* < 0.05, Figure 2C,H,K,N). In contrast, the effects of gefitinib on tumor cells carrying EGFR L858R and ΔE746-A750 mutations were more obvious (*p* < 0.01, *p* < 0.001, Figure 2H,K). Results also showed a significant inhibitory effect on tumor cells harboring the EGFR WT genotype (*p* < 0.001, Figure 2C), but the effects were slightly weaker than those the ATO treatment group (Figure 2C). When arsenic was combined with gefitinib, the inhibitory effects were better than those of a single agent (Figure 2C,H,K,N).

### 3.3. Inhibitory Effects of ATO Intrapleural Administration on NSCLC Tumor Cells

**Figure 3:**
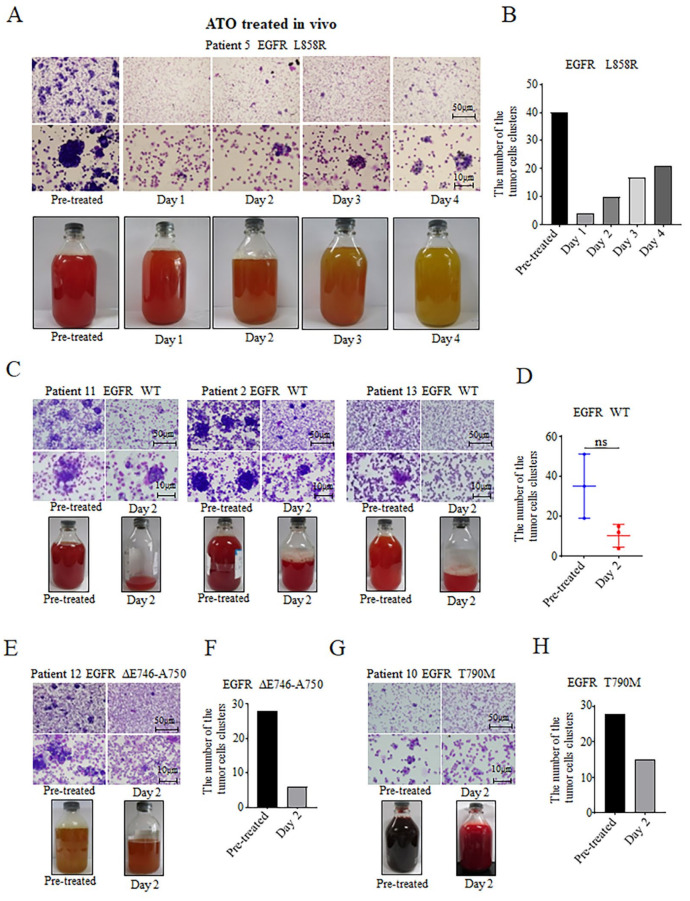
Effects of the arsenic intrapleural administration on tumor cells in pleural effusions from NSCLC patients. (**A**,**B**) Effects of arsenic on tumor cells with EGFR L858R. (**C**,**D**) Effects on pleural effusion and tumor cells with EGFR WT. (**E**,**F**) Effects on pleural effusion and tumor cells with EGFR ΔE746-A750. (**G**,**H**) Effects on pleural effusion and tumor cells with EGFR T790M. Images were captured by an optical microscope. (Scale bars shown on the images). Statistical graphs of the number of tumor cell clusters pre- and after ATO administration are shown in (**B**,**D**,**F**,**H**). “ns” represents “there was no significant difference”.

Patient 5 with the EGFR L858R mutation underwent an intrapleural administration of ATO, and pleural effusions were collected for 4 consecutive days after the administration. Gross photography showed that the color of the pleural effusion gradually changed from bloody to pale yellow with time (Figure 3A). The tumor cells isolated from pleural effusions were stained by Wright’s staining after smearing. It was found that the number of clusters of tumor cells decreased significantly after ATO treatment, with the lowest number on day 1 after treatment, before slightly increasing from day 2 to day 4, but were still significantly lower than before the ATO administration (Figure 3A,B). It was suggested that the inhibitory effects of arsenic on tumor cells are related to the effective concentration of arsenic in the pleural effusion. With the passage of time, the effective concentration of arsenic in the pleural effusion decreases gradually, leading to the increase in the number of tumor cells. In patients with EGFR WT genotype (Patients 11, 2, and 13), the gross photography of pleural effusions after arsenic intrapleural administration showed a significant reduction in the volume of the pleural effusion and the number of clusters of the tumor cells (Figure 3C,D). In Patient 12, who carried an EGFR ΔE746-A750 mutation, intrapleural administration of arsenic reduced the volume of the pleural effusion and the number of tumor cells in the pleural effusion (Figure 3E,F). In Patient 10 with EGFR T790M mutation, the color of the pleural effusion changed from dark red to light red with a decrease in the number of tumor cells (Figure 3G,H). In conclusion, the intrapleural administration of arsenic was able to reduce the amount of the pleural effusion, lighten the color of the pleural effusion, and diminish the number of tumor cells in the pleural effusion in NSCLC patients with various EGFR genotypes.

### 3.4. Effects of Arsenic and Gefitinib on Proliferation and Autophagy in Tumor Cells in Pleural Effusion

**Figure 4:**
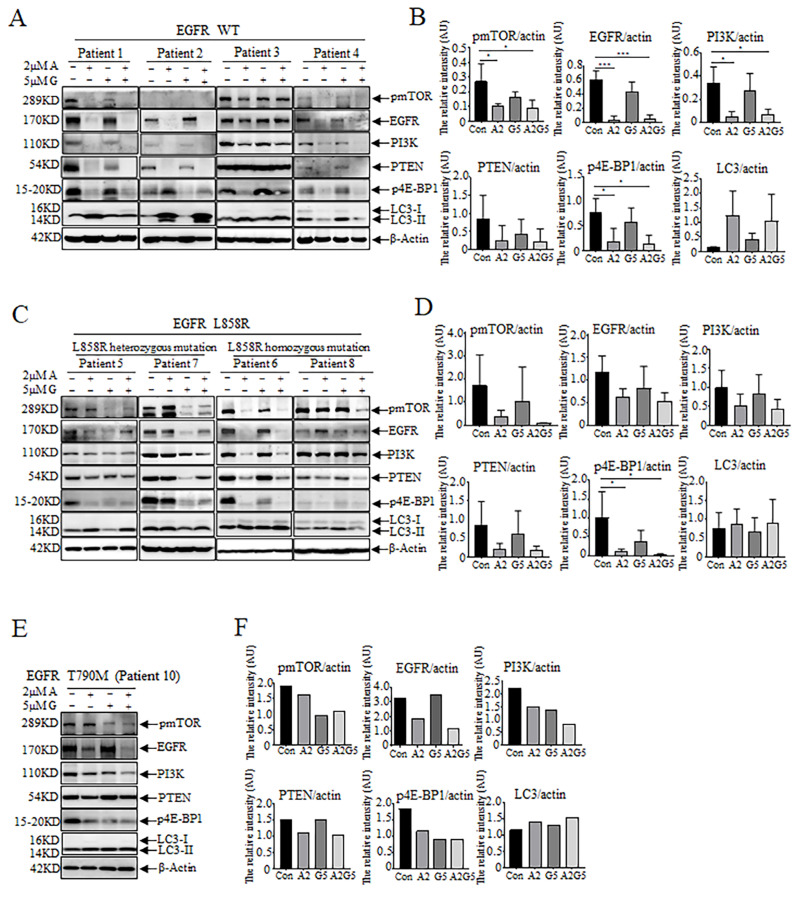
Effects of arsenic and gefitinib on the expression or phosphorylation of EGFR, pmTOR, PI3K, PTEN, p4E-BP1, LC3-II in tumor cells in pleural effusions of NSCLC patients harboring different EGFR genotypes. (**A**,**B**) Effects on tumor cells with EGFR WT. (**C**,**D**) Effects on tumor cells with EGFR L858R. (**E**,**F**) Effects on tumor cells with EGFR T790M. The relative intensity was calculated according to the gray values of pmTOR, EGFR, PI3K, PTEN, p4E-BP1, and LC3-II over that of β-Actin with the Quantity One software (Bio-Rad Laboratories, Inc., Hercules, CA, USA). The results were analyzed by GraphPad Prism version 8.0.1 (GraphPad Software, La Jolla, CA, USA). Pairwise comparisons were performed using Student’s *t* test and two-tailed analysis (* *p* < 0.05, *** *p* < 0.001). “Con” stands for “Control”. “A2” represents “ATO (2 μmol/L) alone”. “G5” stands for “gefitinib (5 μmol/L) alone”. “A2G5” represents “ATO (2 μmol/L) combined with gefitinib (5 μmol/L)”. “AU” represents “arbitrary unit”.

After ATO treatment, the expression of EGFR was decreased not only in tumors cells harboring EGFR WT (Patients 1, 2, 3, 4) (Figure 4A,B), but also in those harboring EGFR L858R mutation (Patients 5, 6, 7, 8) (Figure 4C,D). Additionally, the expression levels of PI3K and PTEN, markers related to tumor proliferation, metastasis, and malignancy degree, were down-regulated. Furthermore, the phosphorylation levels of mTOR and transcription initiation factor eIF4E binding protein (4E-BP1) were also decreased (Figure 4A–D). However, the expression of the autophagy marker LC3-II was up-regulated (Figure 4A–D). This suggests that arsenic can down-regulate the protein expression of EGFR, inhibit proliferation, and promote autophagy. However, in Patient 10, who carries the EGFR T790M resistant mutation, the expression of EGFR was distinctly down-regulated, and the expression of pmTOR, PI3K, PTEN, and p4E-BP1 was slightly decreased, with an indistinct up-regulation of LC3-II after arsenic treatment (Figure 4E,F). In contrast, gefitinib exerted minor effects on the protein expression in tumor cells harboring different EGFR genotypes (Figure 4A–F). At the protein level, the effects of arsenic combined with gefitinib were comparable to or better than those of the arsenic-alone treatment, implying that arsenic may exert an inhibitory effect on NSCLC tumor cells by modulating cell proliferation and autophagy-related proteins at the protein level.

### 3.5. Detection of LC3 and P62 Expression and Distribution in Tumor Cells Derived from Pleural Effusion Treated with Arsenic and Gefitinib by IF

**Figure 5:**
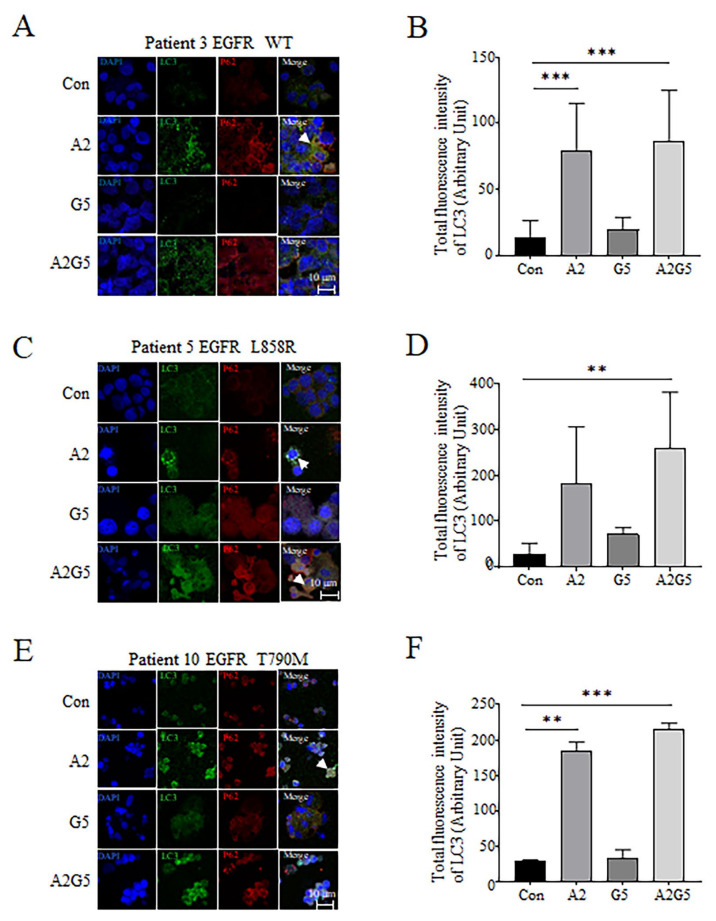
Detection of the expression and distribution of LC3 and P62 in tumor cells with different EGFR genotypes after treatment with arsenic and gefitinib by IF. (**A**) The expression and distribution of LC3 and P62 in tumor cells with EGFR WT. (**B**) The total fluorescence intensity of LC3 was obtained by Bioflux 200 software (Fluxion Bioscience, Oakland, California, USA) and analyzed by GraphPad Prism version 8.0.1. Pairwise comparisons were performed using Student’s t test and two-tailed analysis. (**C**) The expression and distribution of LC3 and P62 in tumor cells with EGFR L858R. (**D**) Statistical graph of the total fluorescence intensity of LC3. (**E**) The expression and distribution of LC3 and P62 in tumor cells with EGFR T790M. (**F**) Statistical graph of the total fluorescence intensity of LC3. White arrows indicate the colocalization of LC3 and P62. Images were captured by a laser confocal microscope, SP8 MP (scale bars, 10 μm). ** *p* < 0.01, *** *p* < 0.001. “Con” stands for “Control”. “A2” represents “ATO (2 μmol/L) alone”. “G5” stands for “gefitinib (5 μmol/L) alone”. “A2G5” represents “ATO (2 μmol/L) combined with gefitinib (5 μmol/L)”.

Using an IF assay, we detected the intracellular expression and distribution of the autophagy marker proteins LC3 and P62 in tumor cells derived from NSCLC patients’ pleural effusion treated with arsenic and gefitinib. Results revealed that arsenic increased the cytosolic expression of LC3 and P62 to a certain extent in tumor cells harboring EGFR WT (Figure 5A,B), EGFR L858R mutation (Figure 5C,D), and T790M mutation (Figure 5E,F). The colocalization of LC3 and P62 can be observed in some regions, as indicated by a white arrow. Arsenic significantly enhanced the expression of LC3 in NSCLC tumor cells (*p* < 0.001, *p* < 0.01, Figure 5B,F), but gefitinib had a weak effect on the expression of LC3 and P62 in tumor cells from patients harboring various EGFR genotypes (Figure 5A–F). The effect of arsenic combined with gefitinib was substantially stronger than that of a single agent (*p* < 0.001, *p* < 0.01, Figure 5B,D,F).

## 4. Discussion

Our previous study showed the effects and mechanisms of arsenic on NSCLC at cellular and animal levels [21], but there are no data regarding the effects of arsenic on tumor cells from NSCLC patients. Therefore, we designed this study to further explore the effects and mechanisms of arsenic on tumor cells in the pleural effusion of patients with NSCLC. In the dose–response analysis, the half maximal inhibitory concentration (IC50) of ATO was 2 μmol/L for NCI-H1975 cells, which is the gefitinib-resistant cell line [21]. Given that ATO at 2 μmol/L is a conventional dosage for leukemia cells [26,27], and is effective on gefitinib-resistant NSCLC cells [21], we thus used ATO at 2 μmol/L to treat tumor cells in the pleural effusion of patients with NSCLC in the present study. The proliferation and viability of three NSCLC cell lines after ATO and/or gefitinib treatment were measured by the Cell Counting Kit-8 (CCK-8) assay and results showed that ATO and gefitinib significantly inhibited the proliferation of NCI-H1975 and HCC827 cells, respectively. In addition, the tyrosine kinase activity of EGFR in NCI-H1975, HCC827, and A549 cells after ATO and/or gefitinib treatment was tested with a tyrosine kinase assay kit, and ATO exhibited a similar inhibition on the three NSCLC cell lines [21]. Based on these data, the present study aimed to further evaluate the effects of arsenic on tumor cells in the pleural effusion of patients with NSCLC, and Wright’s staining was chosen to observe cell morphologies and test the effects of arsenic on tumor cells.

The malignant pleural effusion (MPE) represents a common complication of lung cancer and is associated with poor prognosis [23]. The cell pellet in a pleural effusion can be used for cytological diagnosis and genetic testing. However, the cell composition of pleural effusions is complicated. Typically, there are immune cells, lymphocytes, and neutrophils in the pleural effusion [28]. Specifically, many red blood cells are contained in hemorrhagic pleural effusions [29]. Given the multiple cellular composition of pleural effusions, the lymphocyte isolation solution was applied to isolate mononuclear cells including tumor cells. Although most of the cells isolated by lymphocyte isolation solution were tumor cells, a small number of lymphocytes was still inevitably mixed in the cell pool. To further purify the tumor cells, specific tumor markers might be considered for use in future studies. For the results of *EGFR* genotype identification, specific primers of *EGFR* exon 18, 19, 20, and 21 were used by PCR, and the PCR products were sequenced to ensure the accuracy of the results. However, since Sanger sequencing is unable to detect variants with mutation rates lower than 15% [30], resulting in false negative results of EGFR mutations, more sensitive methods such as next-generation sequencing (NGS) and quantitative PCR (qPCR) are expected to be applied in future studies.

The occurrence of a malignant pleural effusion may be caused by many factors, such as pleural inflammation, tumor angiogenesis, and increased vascular permeability [28,29]. In this study, we found that the color of the pleural effusion in some patients changed from bloody to pale yellow, and the volume of pleural effusion significantly decreased after intrapleural arsenic treatment (Figure 3). It was suggested that the arsenic reduced the pleural vascular permeability, resulting in a decrease in red blood cells in the pleural effusion, which led to a lighter color of the pleural effusion, and in a decrease in the volume of pleural effusions (Figure 3). These results are consistent with previous studies that showed ATO decreased vascular density and permeability [24,25,31].

Results demonstrated that arsenic plays a therapeutic effect at the protein level, since it can degrade the oncoproteins through the ubiquitin-proteasome pathway or via the autophagy pathway [32,33,34]. Our previous study revealed that arsenic inhibited NSCLC not only in cell lines, but also in mice with a subcutaneous tumor and in situ carcinoma [21]. Regarding the mechanism, ATO promoted autophagic degradation of EGFR in NSCLC cells by directly binding to P62, which interacted with EGFR, and preferentially the L858R/T790M mutant, providing an explanation for the more favorable effect of ATO on gefitinib-resistant cells [21].

However, the effects of arsenic on tumor cells in pleural effusions of NSCLC patients are still not completely understood. Here, we found that arsenic decreased the number of clusters of tumors cells isolated from pleural effusions in NSCLC patients with different *EGFR* genotypes (Figure 2). Therefore, we think that arsenic could play roles in patients without *EGFR* mutations. Even in tumor cells harboring T790M (gefitinib-resistant mutant), arsenic was still effective (Figure 2L,N). The results are consistent with our previous studies [21]. Furthermore, WB showed that arsenic down-regulated EGFR expression (Figure 4), consistent with what was found at the cellular level [21]. It was also revealed that arsenic down-regulated the expression or phosphorylation of PI3K, pmTOR, PTEN, and p4E-BP1, and up-regulated the expression of the autophagy-related protein LC3-II (Figure 4).

Previously published data demonstrated that ATO could circumvent the gefitinib resistance by binding to P62 and mediating autophagic degradation of EGFR in NSCLC [21]. We further explored the possible mechanisms of ATO on tumor cells in the pleural effusions of patients with NSCLC in the present study. The PI3K/AKT/mTOR signaling pathway is closely related to tumor survival, proliferation, and distant metastasis [35,36]. The AKT can be phosphorylated by PI3K and mTORC2 to regulate multiple biological functions, such as cell growth, survival, and invasion. The AKT, once activated, may phosphorylate many downstream molecules including mTORC1. Furthermore, mTORC1 is able to phosphorylate the transcription initiation factor eIF4E-binding proteins (4E-BPs), and the phosphorylated 4E-BPs dissociated from eIF4E, and these events promote the entry of eIF4E into the transcription complex for transcription [36]. Recent studies demonstrated that the high expression of pAKT, pmTOR, and peIF4E can be detected in some patients with NSCLC, especially in those with lymph node metastasis, confirming that the high expression of these proteins is positively correlated with the degree of tumor malignancy and with poor prognosis [36,37]. In this study, we found that arsenic down-regulated PI3K and PTEN (Figure 4), which led to the inhibition of AKT phosphorylation and ultimately led to the inhibition of cell growth, proliferation, and invasion. In addition, the down-regulation of pmTOR and p4E-BP1 was also observed (Figure 4), which suggested that 4E-BP1 prevented eIF4E from entering the transcription complex and reduced the transcription of downstream oncogenes. All the results indicate that arsenic inhibited the proliferation, reduced the transcription of oncogenes, decreased the ability of tumor metastasis, and ultimately degraded the malignancy of NSCLC and improved the patients’ prognosis.

Autophagy is a response of cells to starvation or stress, which can achieve homeostasis by removing damaged intracellular proteins and organelles [38]. Autophagy exerts both protective and inhibitory effects on tumors. Autophagy may help tumor cells to maintain mitochondrial function and energy balance under starvation, so that tumors can survive under starvation [39]. On the other hand, in a physiological state, changing the expression of autophagy-related proteins by gene mutation and other means can promote cell death [40]. Therapeutic drugs can exert effects by regulating autophagy, leading to a degradation of oncoproteins and the autophagic death of tumor cells [39].

mTOR, a major modulator of autophagy, plays a switching role in the initiation stage of autophagy. When the activity of mTOR is inhibited, autophagy can start [41]. The mTOR is also a downstream target of the PI3K/AKT pathway [42], which promotes cell growth, differentiation, and survival [43]. Therefore, inactivation of the PI3K/AKT/mTOR pathway would inhibit survival and induce autophagy by mTOR inactivation [42]. PI3K/AKT/mTOR is a classic autophagy pathway [44,45,46]. Studies showed that suppression of the PI3K/AKT/mTOR signaling pathway is a major characteristic of ATO-induced autophagy in NSCLC [44]. LC3 is the major marker of autophagosome, and when intracellular LC3-II is up-regulated, autophagy is induced [47]. As an adaptor protein, P62, is a selective autophagy substrate that may interact with LC3 to mediate the degradation of the proteins bound to P62 through the autophagy pathway [47]. In this study, WB results showed that arsenic induced the down-regulation of PI3K and pmTOR (Figure 4), suggesting that arsenic plays a role in the initiation phase of autophagy. The up-regulation of LC3-II suggested autophagy was promoted by arsenic (Figure 4). The IF results showed that arsenic up-regulated the expression of LC3 and P62, with a co-localization of LC3 and P62 in some cells (indicated by a white arrow) (Figure 5). All the results suggested that arsenic played a therapeutic role in tumor cells from NSCLC patients through the PI3K/AKT/mTOR-mediated autophagic pathway.

In terms of treatment of NSCLC, for several years, systemic chemotherapies including platinum-based regimens have been a mainstay after complete resection. However, after the advent of a variety of new treatment regimens, such as many effective targeted therapies including the third generation EGFR TKIs, as well as immunotherapy, the landscape of lung cancer treatment has been changed [48,49]. ICIs have shown tremendous benefits in the treatment of NSCLC and are now being used as first-line therapies in metastatic disease, consolidation therapy following chemoradiation in unresectable locally advanced disease, and adjuvant therapy following surgical resection and chemotherapy in resectable disease [50].

We think it is necessary to compare the efficacy, mechanisms, and safety of targeted therapy (TKIs), chemotherapy, immunotherapy (ICIs), arsenic, surgery, and radiation therapy in NSCLC (Table 2). With the development of biomarkers, the treatment of lung cancer has also evolved with the introduction of several lines of TKIs in patients with *EGFR*, *ALK*, *ROS1*, and *NTRK* mutations [8]. Among them, gefitinib is the first approved agent targeting the tyrosine kinase (TK) of EGFR. A large-scale clinical study showed that gefitinib provided a striking antitumor activity for lung adenocarcinoma with EGFR-activating mutations [51,52]. Although most *EGFR*-mutated NSCLC patients initially respond to gefitinib, patients usually develop acquired resistance (T790M) [51]. To further improve the therapeutic outcomes, more potent EGFR-TKIs through irreversible inhibition of tyrosine kinase (osimertinib) have been developed [51]. Osimertinib, as a third-generation EGFR-TKI that selectively inhibits mutated-EGFR through irreversible and covalent binding to C797 in the TK domain [53], has shown more potent inhibition of TK activity [54] and a superior PFS (progress free survival) over gefitinib [55]. For advanced NSCLC harboring *EGFR* mutations, EGFR-TKIs are preferably prescribed as they provide a superior survival benefit over chemotherapy [51]. In a phase 3 trial comparing osimertinib with chemotherapy for patients who developed acquired resistance caused by T790M, osimertinib showed a superior PFS over chemotherapy [14].

Platinum-based chemotherapy drugs are widely used in human neoplasms [58]. NSCLC patients are less sensitive to platinum-based chemotherapy including cisplatin and carboplatin [59]. The mechanisms of the therapeutic and toxic effects of platinum drugs on cells are involved as a consequence of the covalent adduct formation between platinum complexes and DNA, RNA, and proteins [14]. The major obstacle for the clinical use of platinum-based anticancer drugs is the development of resistance and toxicity [58].

Immunotherapy with ICIs, which unleash a patient’s own T cells to kill tumors, has become a “game changer” in the treatment of advanced NSCLC. The most clinically important advantage over chemotherapy is its long-term survival benefits [60,71]. ICIs may restore the ability of cancer immunity to kill tumor cells by blocking some inhibitory molecules of “immune checkpoints” such as PD-1, PD-L1, and cytotoxic T-lymphocyte-associated protein 4 (CTLA-4) [60]. Nevertheless, the superiority of ICIs in advanced EGFR-mutant NSCLC is only moderate. A non-negligible proportion of patients experience an ultrarapid disease progression [72,73]. Resistance to ICIs restricts the number of patients able to achieve durable responses, and immune-related adverse events may complicate the treatment [61,62].

Surgery is currently the standard treatment for patients with stage I NSCLC. Radiation therapy, stereotactic body radiation therapy (SBRT) in particular, is recommended for those who are not medically fit for surgery [69]. Radiotherapy can take effect via inducing DNA damage and the generation of reactive oxygen species (ROS) to destroy cancer cells and tumor tissue [70]. The development and improvement of minimally invasive interventional techniques, such as SBRT, percutaneous ablation, and bronchial intervention, provide more options for lung cancer treatment. Each treatment has its own unique advantages, but also has corresponding limitations. The combination of a variety of treatments may be able to exert the best therapeutic effects. For example, SBRT, RFA (radiofrequency ablation), MWA (microwave ablation), CRA (cryoablation), and other therapeutic methods have shown the possibility of combined application with novel treatments including immunotherapy [74].

The therapeutic efficacy and safety of arsenic in clinical trials has been well demonstrated in the treatment of leukemia. The 5-year DFS rate of APL after treatment with ATO combined with ATRA is more than 95% [19]. The long-term safety of arsenic has been evaluated. Generally, the toxicity profile was found to be mild and reversible [20]. The tolerable and reversible grade I-II liver dysfunction could be detected. There were no abnormal electrocardiograms and echocardiograms that might be associated with the long-term toxicity of ATO. Results of laboratory and systemic physical examination were comparable between patients and healthy donors. Moreover, arsenic concentrations in the urine of patients who had ceased ATO treatment for 24 months were below the safety limits, whereas those in plasma, nails, and hair were only slightly higher than those found in healthy controls [20]. Nevertheless, pleural and/or pericardial effusions in patients undergoing ATO treatment have been reported in individual case reports [67,68]. After 2 weeks of ATO therapy suspension there was evidence of a complete resolution of pleural and pericardial effusions [67]. In the treatment of pleural effusions in NSCLC, the overall remission (OR) rate of arsenic is 70.8%, that of complete remission (CR) is 8.3%, and that of partial remission (PR) is 62.5% [25]. It is suggested that arsenic could potently reduce the volume of pleural effusions when maintained for more than 1 month. The mild adverse effects include gastrointestinal reactions, fever, chest pain, and leukopenia. The symptoms resolved spontaneously without medication [24,25].

Gefitinib [56] and osimertinib [57] demonstrated a relatively weak reversal of chemotherapy resistance. In contrast, arsenic trioxide played roles in treatment of cisplatin-resistant NSCLC PC-9/CDDP and PC-14/CDDP cells [63]. Arsenic sulfide could reverse cisplatin resistance in NSCLC in vitro and in vivo through targeting PD-L1 [22]. Arsenic might sensitize NSCLC cells to cisplatin through targeting the p53/miR-34a-5p/PD-L1 axis. In addition, arsenic trioxide exerted synergistic effects with cisplatin on NSCLC via apoptosis induction [64]. ATO combined with gefitinib demonstrated better inhibitory effects than those of a single agent, both on NSCLC cell lines [21] and on tumor cells from pleural effusions in NSCLC patients (Figure 2 and Figure 5). Moreover, arsenic could also circumvent the gefitinib resistance by binding to P62 and mediating autophagic degradation of EGFR in NSCLC [21]. Arsenic can induce apoptosis and arrest the cell cycle in lung cancer cells [65]. Additionally, ATO inhibited the growth of lung cancer xenograft tumors and the formation of malignant pleural effusion in a mice model because of its antiangiogenic effects [31]. Arsenic trioxide inhibited cancer stem-like cells via down-regulation of Gli1 in lung cancer [66]. Given that the mechanisms of arsenic are different from those of other strategies, we theoretically speculate that arsenic has the potential to be used in combination with all therapeutic strategies, such as TKIs, ICIs, chemotherapy, and radiotherapy, in all tumor stages, but the exact effects need to be evaluated in future clinical trials.

To sum up, targeted drugs and ICIs are gaining increasing importance in the treatment of NSCLC and these novel treatments will supplement chemotherapy, radiotherapy, and surgery. For gefitinib-sensitive and -resistant NSCLC, TKIs might exert potently therapeutic effects. In contrast, in all stages of NSCLC, platinum-based adjuvant chemotherapy could play a synergistic therapeutic role. However, for NSCLC resistant to TKIs and platinum-based chemotherapy drugs, arsenic could still be effective. Therefore, utilization of arsenic to treat NSCLC is a promising and potential therapeutic strategy.

However, this study has limitations, including the limited number of NSCLC samples, especially of the gefitinib-resistant NSCLC samples. In addition, other proteins of the PI3K/AKT/mTOR pathway were not assessed, and the sensitivity of Sanger sequencing in the detection of gene mutations was low. These limitations will be addressed in future studies.

## 5. Conclusions

In conclusion, for NSCLC patients with pleural metastasis and pleural effusions, intrapleural administration with arsenic has a certain therapeutic effect and significance, making it suitable as a therapeutic strategy for these patients. The findings provide important preliminary data that can be used for future development of strategies using arsenic combined with TKIs, chemotherapy, and immunotherapy, and even with surgery and radiation therapy, for the treatment of NSCLC in clinical trials.

## Figures and Tables

**Table 1:** Sheet of basic information for NSCLC patients.

Clinical Character (*n* = 36)	Number (%)
Gender (%)	
Female	13 (36.1)
Male	23 (63.9)
Age (median [Q1; Q3])	63.5 [54.0; 73.0]
Smoking (%)	
Never	19 (52.8)
Former	3 (8.33)
Current	14 (38.9)
Type (%)	
Adeno-carcinoma	32 (88.9)
Poorly differentiated adeno-carcinoma	4 (11.1)
T stage (%)	
T1	2 (5.56)
T2	9 (25.0)
T3	8 (22.2)
T4	17 (47.2)
N stage (%)	
N1	6 (16.7)
N2	12 (33.3)
N3	18 (50.0)
M stage (%)	
M1a	17 (47.2)
M1b	16 (44.4)
M1c	3 (8.33)
Appearance of pleural effusions (%)	
Yellow and muddy	17 (47.2)
Bloody and muddy	19 (52.8)
EGFR Genotype (%)	
WT	25 (69.4)
L858R	6 (16.7)
T790M	2 (5.56)
ΔE746-A750	3 (8.33)

**Table 2:** Comparation of the efficacy, mechanisms, and safety of targeted therapy (TKIs), chemotherapy, immunotherapy (ICIs), arsenic, surgery, and radiation therapy on NSCLC.

Therapies	Efficacy						Mechanisms	Side Effects
	EGFR WT	EGFR L858R or ΔE746-A750	EGFR T790M	PD-1/PD-L1	Chemo- Resistance	PFS/OR/OS		
**TKIs**								
Gefitinib	− [51,52]	++ [51,52]	− [51,52]		+/− [56]	OR 71.2% in NSCLC with EGFR mutations [51], 12-month PFS 24.9% [52].	Inhibition of the EGFR tyrosine kinase activity by binding to ATP pocket [16,51].	Rash or acne, diarrhea, dry skin, anorexia, pruritus, stomatitis, asthenic conditions, nausea, paronychia, vomiting, constipation, neurotoxic effects, myalgia, arthralgia [52]
Osimertinib	− [54]	+ [54]	+++ [51,53,54]		+/− [57]	PFS 10.1 months [14,51], PFS 18.9 months in a first-line treatment in the FLAURA trail [51].	Selective inhibition mutant EGFR through irreversible and covalent binding to C797 in the tyrosine kinase domain [51,54].	Diarrhea, rash, dry skin, paronychia [14].
**Chemotherapy**								
Cisplatin/ Pemetrexed	+/− [58,59]	+/− [51,52]	+/− [58,59]		− [58,59]	PFS 4.4 months [14,51].	Formation of covalent adducts between platinum complexes and DNA, RNA, and proteins [58,59].	Nausea, decreased appetite, constipation, anemia [14].
Carboplatin /Paclitaxel	+/− [51,52]	+/− [51,52]	+/− [51,52]		− [58,59]	12-month PFS 6.7% [52].	Formation of covalent adducts between platinum complexes and DNA, RNA, and proteins [58,59].	Rash or acne, diarrhea, dry skin, anorexia, pruritus, stomatitis, asthenic conditions, nausea, paronychia, vomiting, constipation, neurotoxic effects, myalgia, arthralgia [52].
**ICIs**								
Nivolumab (anti-PD-1)	+/− [60]	+/− [60]	+/− [60]	++ [60]	+ [61]	OR 20%, PFS 3.5 months, OS 9.2 months [60].	Restore the ability of cancer immunity to kill tumor cells by blocking PD-1 [60].	Pneumonitis, hepatitis, neurotoxic effects, myocarditis, toxicity-related fatality rates is 0.36% [62].
Pembrolizumab (anti-PD-1)	+/− [60]	+/− [60]	+/− [60]	++ [60]	+ [61]	OR 18%, PFS 3.9 months, OS 10.4 months [60].	Restore the ability of cancer immunity to kill tumor cells by blocking PD-1 [60].	Pneumonitis, hepatitis, neurotoxic effects, myocarditis, toxicity-related fatality rates is 0.36% [62].
Durvalumab (anti-PD-L1)	+/− [60]	+/− [60]	+/− [60]	++ [9,50]	+ [9,50]	Median PFS 16.8 months [9], 12-month PFS 55.9%, 18-months PFS 44.2 [9].	Restore the ability of cancer immunity to kill tumor cells by blocking PD-L1 [60].	Diarrhea, pneumonitis, rash, pruritus, cough, fatigue, dyspnea, pyrexia, decreased appetite, nausea, arthralgia, constipation, anemia [9].
Atezolizumab (anti-PD-L1)	+/− [60]	+/− [60]	+/− [60]	++ [60]	+ [61]	OR 14.6%, PFS 2.7 months, OS 12.6 months [60].	Restore the ability of cancer immunity to kill tumor cells by blocking PD-L1 [60].	Pneumonitis, hepatitis, neurotoxic effects, myocarditis, toxicity-related fatality rates is 0.38% [62].
**Arsenic**								
ATO	+ [this study]	+ [21,this study]	++ [21,this study]		+ [63]	5-year DFS > 95% in APL [19] To treat pleural effusion in NSCLC, OR 70.8%, CR 8.3%, PR 62.5% [25].	Degradation of the oncoproteins [21]; inhibition proliferation [21,64]; induction apoptosis [64] and autophagy [21]; cell cycle arrest [65]; reduction pleural vascular permeability [31]; inhibition of the cancer stem-like cells [66]; reversion cisplatin resistance [63].	Tolerable and reversible grade I-II liver dysfunction [20]; reversible pleural and/or pericardial effusion [67,68]; mild gastrointestinal reactions, fever, chest pain, leukopenia [24,25].
Arsenic sulfide				+ [22]	+ [22]		Sensitize NSCLC cells to cisplatin through targeting PD-L1 [22].	
**Surgery**								
Lobectomy Wedge resection Segmentectomy	The standard treatment for patients with stage I NSCLC [69].					3-year OR 82%, 5-year OR 66% [69].	Lobectomy, wedge resection, segmentectomy can remove the lesion [69].	Recurrence, pneumonia, respiratory failure, myocardial infarction [69].
**Radiotherapy**								
SBRT	SBRT is recommended for those who are not medically fit for surgery. SBRT is extremely well tolerated. SBRT is an outpatient procedure [69].					2-year OR 84%, 3-year OR 77%, 5-year OR 55.7%, 7-year OR 47.5% [69], Local tumor control rates > 90% [69].	Induce DNA damage, terminate cell division and proliferation, lead to cell necrosis and apoptosis. Induce the generation of ROS, which can induce cellular stress in, and injure biomolecules, alter cellular signaling pathways [70].	Mild fatigue, exacerbate degenerative arthritis in the shoulders, back, hips. Decreased pulmonary function. Chest pain, rib fractures. Esophagitis, skin irritation, brachial plexopathy [69].

“+” means effective, “−” means ineffective, “+/−” represents a weak effect.

## Data Availability

The data that support the findings of this study are available from the corresponding author upon reasonable request.
